# Prediction of Congenital Portosystemic Shunt in Neonatal Hypergalactosemia Using Gal-1-P/Gal Ratio, Bile Acid, and Ammonia

**DOI:** 10.3390/ijns11030061

**Published:** 2025-08-07

**Authors:** Sayaka Suzuki-Ajihara, Ikuma Musha, Masato Arao, Koki Mori, Shunsuke Fujibayashi, Ihiro Ryo, Tomotaka Kono, Asako Tajima, Hiroshi Mochizuki, Atsuko Imai-Okazaki, Ryuichiro Araki, Chikahiko Numakura, Akira Ohtake

**Affiliations:** 1 Department of Pediatrics, Saitama Medical University, Saitama 350-0495, Japan; sayaka31849@gmail.com (S.S.-A.); musha@saitama-med.ac.jp (I.M.); masato_a@saitama-med.ac.jp (M.A.); cnumasmu@gmail.com (C.N.); 2 Department of Clinical Genomics, Saitama Medical University, Saitama 350-0495, Japan; 3 Department of Clinical Laboratory, Saitama Children’s Medical Center, Saitama 330-8777, Japan; sc.mass-screening@saitama-pho.jp; 4 Division of Endocrinology and Metabolism, Saitama Children’s Medical Center, Saitama 330-8777, Japan; fujibayashi.shunsuke@saitama-pho.jp (S.F.); ryo.ihiro@saitama-pho.jp (I.R.); k_tomoro@ymail.ne.jp (T.K.); atajima24@gmail.com (A.T.); hmochi300514@gmail.com (H.M.); 5 Diagnosis and Therapeutics of Intractable Diseases, Intractable Disease Research Center, Juntendo University Graduate School of Medicine, Tokyo 113-8421, Japan; a-okazaki@juntendo.ac.jp; 6 Medical Education Center, Saitama Medical University, Saitama 350-0495, Japan; raraki@saitama-med.ac.jp; 7 Center for Intractable Diseases, Saitama Medical University Hospital, Saitama 350-0495, Japan

**Keywords:** congenital portosystemic shunt, hypergalactosemia, newborn screening, galactose-1 phosphate/galactose, total bile acid, ammonia

## Abstract

Congenital portosystemic shunts (CPSSs) are often associated with life-threatening systemic complications, which may be detected by identifying hypergalactosemia in newborn screening (NBS). However, diagnosing CPSS at an early stage is not easy. The purpose of this study was to predict CPSS early using screening values and general blood tests. The medical records of 153 patients with hypergalactosemia who underwent NBS in Saitama Prefecture between 1 December 1997 and 31 October 2023 were retrospectively analyzed. We provided the final diagnosis of the analyzed patients. Of the 153 patients, 44 (29%) were in the CPSS group and 83 (54%) were in the transient galactosemia group. Using the initial screening items and the six blood test items, we attempted to extract a CPSS group from the transient galactosemia group. Finally, a model for CPSS prediction was established. From multiple logistic regression analysis, filtered blood galactose-1 phosphate/galactose, serum total bile acid, and ammonia were adopted as explanatory variables for the prediction model. If the cut-off value for predicted disease probability value (P) was >0.357, CPSS was identified with 86.4% sensitivity (95%CI 72.6–94.8%) and 81.9% specificity (95%CI 72.0–89.5%). This predictive model might allow prediction of CPSS and early intervention.

## 1. Introduction

Galactosemia is an inborn error of galactose metabolism. This pathology has been included in various neonatal screening programs since 1977 in Japan. The enzyme deficiency can be detected by hypergalactosemia. However, causes of hypergalactosemia also include congenital portosystemic shunt (CPSS), citrin deficiency, and transient galactosemia [1].

CPSS is a rare congenital malformation, occurring in 1 in 30,000 newborns [2]. Several reports from Japan have described CPSS as a frequent cause of hypergalactosemia identified from newborn screening [3].

CPSS can lead to various complications such as hyperammonemia, neurological complications, hepatic encephalopathy, liver tumors, and hepatopulmonary syndrome. Shunts can be categorized into two kinds: intrahepatic, which largely resolve spontaneously; and extrahepatic, which usually require surgical correction or, in some cases, liver transplantation [4]. Therefore, depending on the shunt type and size, intervention before symptoms appear may be desirable [5].

The diagnosis of CPSS requires imaging. Ultrasonography (US) is the modality of choice for every patient based on its ease of use, utility, non-invasiveness, and the ability to avoid radiation exposure. However, CPSS may be missed on US due to limited imaging features and interference from gastrointestinal gas [6,7,8]. To compensate for this insufficient ability of US, a study in Japan reported that using filtered blood galactose (Gal), galactose-1 phosphate (Gal-1-P), serum total bile acid (TBA), and blood ammonia (NH3) enabled detection of CPSS [8], although the number of cases under investigation was small and no similar studies have been reported.

The purpose of this study was to determine the utility of differentiating CPSS using screening values and general blood tests.

## 2. Materials and Methods

### 2.1. Newborn Screening

This retrospective study was conducted in the Division of Mass Screening at Saitama Prefectural Children’s Medical Center, from 1 December 1997 to 31 October 2023.

Dried blood spots (DBS) were collected approximately 1–2 h after feeding between 4 and 6 days after birth. Screening parameters included Gal, total Gal (TGal), Gal-1-P, UDP-galactose-4-epimerase (GALE) activity, and galactose-1-phosphate uridylyltransferase (GALT) activity. Gal and Gal-1-P were measured using a microplate enzyme assay method [9]. Values before the addition of alkaline phosphatase (ALP) were taken as Gal, while values after the addition of ALP were taken as TGal. Gal-1-P was calculated as [TGal − Gal] × 1.44. The activities of GALT and GALE were measured using spot test and fluorescence detection methods [10,11]. GALE activity measurements began from 2013.

### 2.2. Cut-Off Values

The cut-off values for immediate confirmatory testing were set at Gal ≥ 10 mg/dL or TGal ≥ 20 mg/dL from 1997 to the end of 2012, and Gal ≥ 10 mg/dL or Gal-1-P ≥ 20 mg/dL from the start of 2013. The Gal-1-P cut-off was raised to ≥ 25 mg/dL in August 2018 to improve screening specificity. Newborns with Gal ≥ 3 mg/dL or TGal ≥ 7 mg/dL in 1997–2003, with Gal ≥ 3 mg/dL or TGal ≥ 8 mg/dL in 2004–2012, or with Gal ≥ 3 mg/dL or Gal-1-P ≥ 15 mg/dL after 2013 underwent retesting. In this study, to standardize the timing of testing for all cases, values from the initial newborn screening were used.

### 2.3. Confirmatory Testing

Confirmatory blood testing included alanine aminotransferase (ALT), albumin, blood ammonia (NH3), direct bilirubin, prothrombin time (PT%), TBA, and plasma amino acid analysis. These values were collected approximately 1–2 h after feeding. Abdominal ultrasonography was performed as an imaging examination, and contrast-enhanced computerized tomography (CT) was performed for consenting patients.

### 2.4. Handling for Hypergalactosemia

When blood galactose level was ≥10 mg/dL, we basically used lactose-free formula. Genetic testing was considered when each of *GALT* deficiency, systemic *GALE* deficiency, or citrin deficiency was suspected [12]. If the cause of galactosemia could not be identified, we performed a galactose loading test (normal formula load test) at 2–3 months after birth [13]. If the Gal level increased after loading, genetic testing for galactose mutarotase (*GALM*) deficiency was considered after its discovery in 2018 [13]. All other cases were carefully followed-up as transient galactosemia.

### 2.5. Definitions of CPSS and Transient Galactosemia

Cases of CPSS were defined as those cases showing vascular malformation on imaging. Cases of transient galactosemia were defined as those cases for which causes of galactosemia were unclear. This group might have included cases with delayed closure of the ductus venosus or heterozygous enzyme deficiency.

### 2.6. Types of CPSS

Various shunt classifications have been reported [14,15,16]. In this study, malformation of an intrahepatic portal vein or of a portal vein that does not flow into the liver were categorized as extrahepatic shunt. Cases with a shunt vessel between the intrahepatic portal vein branch, the hepatic vein or inferior vena cava, or patent ductus venous (PDV) were defined as intrahepatic shunt.

### 2.7. Statistical Analysis

To predict CPSS, we statistically compared screening items and confirmatory blood testing for the two groups of CPSS and transient galactosemia.

The unpaired *t*-test, Mann–Whitney U test, and Fisher’s exact test were employed to compare 12 variables between patients in the CPSS and transient groups. Values of *p* < 0.05 were considered statistically significant. Receiver operating characteristic (ROC) analyses were performed to evaluate the ability to predict CPSS. Using these ROC analyses, we determined the cut-off values that yielded the highest Youden index [17]. To correlate Gal-1-P/Gal, TBA, and NH3 with significant CPSS, we applied multiple logistic regression analyses with variable selection by backward elimination. All analyses were performed using SAS JMP Pro version 16.2.0 (SAS Institute Inc., Cary, NC, USA), EZR version 1.64 [18], and MedCalc version 22.030 (MedCalc Software, Mariakerke, Belgium). In this paper, “P” and “*p*-value” denote the predicted disease probability estimated with our clinical prediction model and the statistical probability value obtained with statistical tests, respectively. Internal validation of models was carried out by correcting measures of predictive performance for “optimism” or overfit using bootstrap methods in the rms packages (version 6.7-1) in R using 1000 resamplings [19,20].

## 3. Results

**Figure 1:**
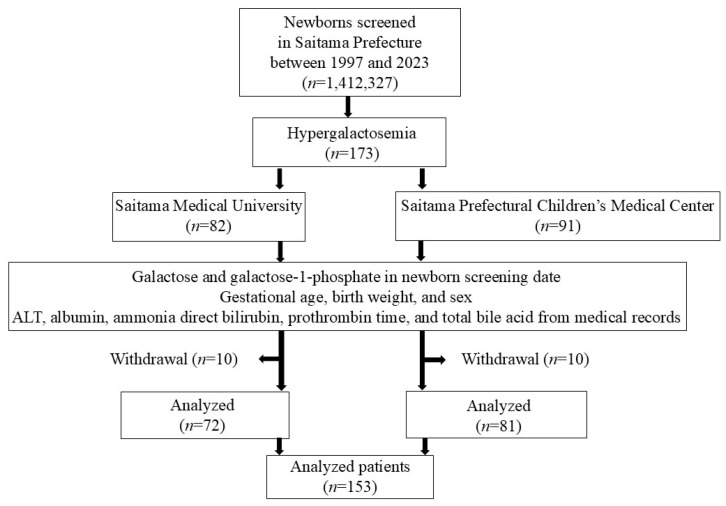
Flow diagram of patients analyzed in this study.

In total, 1,412,327 blood samples from newborns from 1 December 1997 to 31 October 2023 were tested, while 173 cases (0.012%) of hypergalactosemia came to two hospitals (Saitama Medical University Hospital or Saitama Prefectural Children’s Medical Center) for confirmatory testing. After excluding patients with missing values, a final total of 153 cases screened after 2002 were analyzed (Figure 1).

For cases with a final diagnosis of hypergalactosemia, 18 cases (12%) showed enzyme deficiency including heterozygotes, 44 cases (29%) were CPSS, 7 cases (5%) were citrin deficiency, 83 cases (54%) were transient cases, and 1 case (1%) involved biliary atresia. In the cases of enzyme deficiency, *GALE* deficiency (peripheral type) accounted for the largest number of cases (11 cases). All cases of *GALT* deficiency were heterozygous. *GALM* deficiency was discovered in 2018, with cases before that time potentially being classified to the transient group. Cases of CPSS comprised 32 cases (73%) of intrahepatic shunt and 12 cases (27%) of extrahepatic shunt, with intrahepatic shunts being significantly more common (Table 1). Abdominal CT was performed in 15 cases of CPSS and in 29 cases of the transient group, respectively.

Most cases of CPSS closed spontaneously during the first 2 years of life. Surgical intervention was required in 13 of 44 CPSS patients (30%; Table 2). Of these 13 cases, 10 were extrahepatic and 3 intrahepatic, with extrahepatic shunts being more common (*p*-value < 0.001). For extrahepatic CPSS, pre-emptive closure is the consensus even for asymptomatic patients, as spontaneous closure is considered unlikely and this type is associated with more severe complications [4]. For this reason, surgical interventions were performed for 10 of the 12 extrahepatic shunts. Except in one case for which the details were unknown, surgical intervention was performed for four symptomatic and eight asymptomatic cases. Even in asymptomatic cases, procedures for closing shunts were applied if closure was not confirmed by about 2 years old (Table 2). After shunt closure, TBA and NH3 data in almost all cases were improved (Table S1)

Comparing CPSS and transient cases using screening items and confirmatory blood tests, significant differences were identified in Gal, TGal, Gal-1-P, Gal-1-P/Gal, albumin, NH3, and TBA (*p*-value < 0.05 each) (Table 3).

To avoid multicollinearity, Gal-1-P/Gal was selected from the ROC results out of Gal, TGal, Gal-1-P, and Gal-1-P/Gal. Repeated multiple logistic regression analysis was performed using Gal-1-P/Gal and other variables, selecting explanatory variables for the predictive model by backward elimination (Table 4). Finally, Gal-1-P/Gal, TBA, and NH3 were selected as explanatory variables that were statistically significant and used in the predictive model equation [20]:

$$P=\frac{1}{1+e^{\left(1.9398783+0.1090073\times \left[\frac{Gal-1-P}{Gal}\right]-0.01543895\times \left[TBA\right]-0.01569756\times \left[NH3\right]\right)}}$$

**Figure 2:**
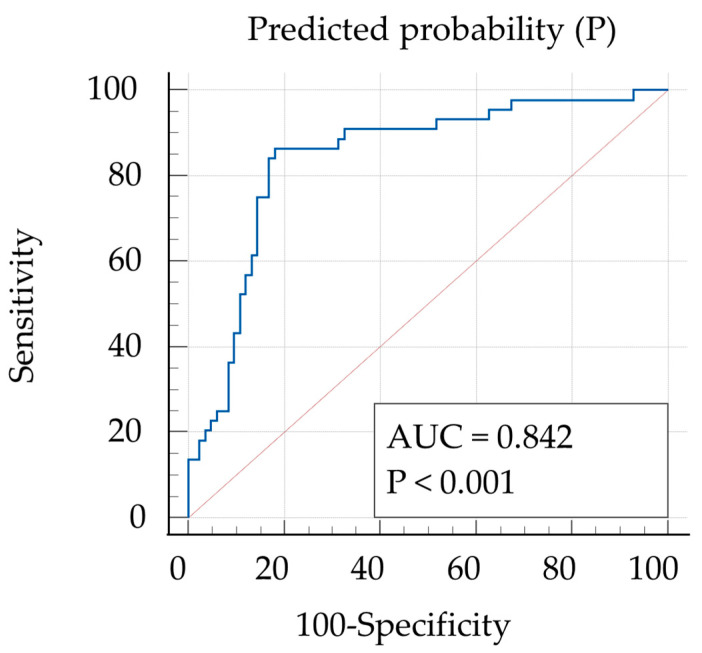
Receiver operating curve (ROC) for predicted probability of congenital portosystemic shunt (CPSS).

**Figure 3:**
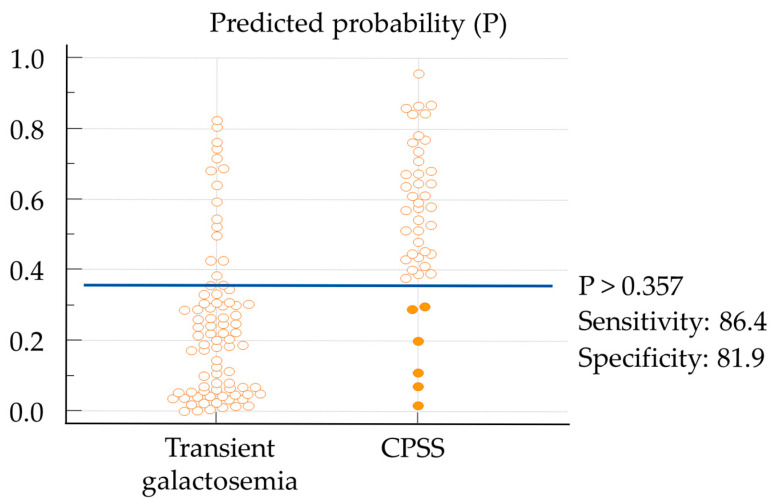
Results of using P > 0.378 for the CPSS group and transient group.

The ROC of the prediction equation (Figure 2) showed that the highest value of the Youden index (area under the curve [AUC] = 0.842) was seen at P = 0.357. Using P > 0.357 as the cut-off, sensitivity was 86.4% (95% confidence interval [CI] 72.6–94.8%) and specificity was 81.9% (95%CI 72.0–89.5%). Values of P could be changed considering sensitivity and specificity (Table S1). Figure 3 presents the results of applying P > 0.357 for the CPSS and transient groups. Most cases of CPSS could be suspected using P > 0.357. Five cases of CPSS showed false-negative results. Four of these cases involved delayed closure of the PDV, with all having high initial Gal-1-P levels (Figure 3). Validation of the prediction model was carried out using the bootstrap method. The AUC for the ROC was 0.832 (bootstrap optimism-corrected c = 0.665), and the optimism-corrected calibration slope was 0.9. The mean absolute error was 0.056, showing good fitness of the model.

The ROC of the prediction equation was calculated using filtered blood galactose-1 phosphate, blood total bile acid, and ammonia as statistically significant explanatory variables for predicting CPSS.

Most cases of CPSS could be suspected using P > 0.357. Six cases of CPSS (orange filled circles) showed false-negative results. If P > 0.357 was used as the cut-off value for predicted disease probability, CPSS was identified with 86.4% sensitivity (95%CI 72.6–94.8%) and 81.9% specificity (95%CI 72.0–89.5%). Figure S1 shows the distribution of the three individual parameters (G1P/Gal ratio, TBA, and NH3) separately.

We showed two representative cases. Case 12 in Table 2 is an example of us being able to close the shunt during the asymptomatic period with the help of our prediction model. She was born to non-consanguineous parents at 40 weeks, 6 days of gestation. Her birth weight was 2170 g (0.1%ile) and height was 45.5 cm (0.7%ile). Phototherapy was performed at seven days due to neonatal jaundice. The initial newborn screening results were Gal 7.2 mg/dL, TGal 15.7, and Gal-1-P 12.2 mg/dL. As Gal was ≥ 3 mg/dL, the patient underwent retesting. The results were Gal 4.2 mg/dL, TGal 8.9, and Gal-1-P 6.8 mg/dL, so confirmatory testing was carried out. The Gal-1-P/Gal, TBA, and NH3 levels were 1.69, 150.6 μmol/L, and 102 μg/dL, respectively. Prediction probability (P) was 0.86. No shunt vessel was detected on abdominal ultrasound, whereas intrahepatic shunt of the portal vein to left hepatic vein was identified on contrast-enhanced CT. She underwent endovascular closure with coils at 1 year and 8 months old because, although she was asymptomatic, the shunt was considered unlikely to close.

The second case required surgical treatment despite a low *p*-value of 0.30 (Case 6 in Table 2). Case 6 was the second child born to non-consanguineous parents, delivered at 39 weeks and 1 day of gestation. His birth weight was 3300 g and height was 50.5 cm; Apgar scores were 2 and 4 at 1 and 5 min, respectively; and the patient was admitted to the neonatal intensive care due to neonatal asphyxia. The initial newborn screening results were as follows: Gal 3.4 mg/dL, TGal 12.8, and Gal-1-P 13.5 mg/dL. As Gal was ≥ 3 mg/dL, the patient underwent retesting. The results were Gal 5.1 mg/dL, TGal 8.1, and Gal-1-P 4.2 mg/dL, so confirmatory testing was carried out. Gal-1-P/Gal, TBA, and NH3 were 3.97, 28.3 μmol/L, and 68.12 μg/dL, respectively. P was 0.30, revealing a false-negative result. The abdominal ultrasound showed an extrahepatic shunt of the splenic vein to the left renal vein. The patient underwent vascular ligation at the age of 3 years, 8 months because, although he was asymptomatic, the shunt remained open.

## 4. Discussion

Galactosemia type 1 was first described in 1908 [21]. After that, with the intention of preventing disease by restricting galactose intake from the onset of symptoms, the NBS began to measure galactose from DBS [22,23]. However, congenital enzyme deficiency has become recognized as uncommon in hypergalactosemia [1]. In this study, enzyme deficiency accounted for only 12% of cases. CPSS was the most common, accounting for 29% of cases, excluding transient cases (Table 1).

The overall incidence of CPSS is estimated to be 1:30,000~40,000 births, and 1:50,000 for cases that persist beyond early life [2]. In this study, the prevalence of CPSS was 1 in 32,098, similar to previous reports [2]. Reports from Japan have examined the frequency of CPSS in hypergalactosemia, varying from 7% to 43% depending on the cut-off value, screening items, and number of cases [3,24]. Taken all together, cases of CPSS in Japan appear to have been mostly detected during NBS.

CPSS patients may present with hypoglycemia, hyperammonemia, and jaundice in the neonatal period, and are at risk of pulmonary hypertension, hepatic encephalopathy, and liver tumors later in life [4]. Uchida et al. reported that the main complications related to CPSS were hyperammonemia (85.2%), liver masses (25.4%), hepatopulmonary shunts (13.9%), and pulmonary hypertension (11.5%). Shunt closure improved most symptoms, except liver masses and pulmonary hypertension. Further, more than half of CPSS patients were detected by NBS [25]. Similar reports have described CPSS detection by the NBS [3,24,25,26,27]. Based on the above results, suspecting CPSS at the time of NBS is important.

CPSS is diagnosed by imaging tests. Non-invasive abdominal ultrasound is the initial imaging modality for diagnosing CPSS, but it may not accurately demonstrate the associated intra- or extrahepatic shunts due to factors such as limited imaging features and gastrointestinal gas interference [6]. In such cases, contrast-enhanced CT or MRI with radiation exposure needs to be considered. In this study, ultrasound was performed in all cases; several cases of CPSS were missed on US but detected on contrast-enhanced CT. CPSS is thus not easily diagnosed and may be missed [6,7,8]. When the cut-off value for predicted disease probability (P) was set at 0.357 or higher, 14 out of 15 cases diagnosed by CT in the CPSS group were positive, with only one false negative. This indicates that the number of false negatives decreased when CT was used for diagnosis in the CPSS group (Figure S2). For limitation, the possibility that some cases in the CPSS who did not undergo CT imaging could represent false negatives if the abdominal ultrasound failed to detect a shunt.

Regarding CPSS treatment, intrahepatic CPSS diagnosed at birth or in utero is generally recommended to be monitored for spontaneous closure during the first 2 years of life, provided no significant clinical complications arise. If the shunt does not close spontaneously and remains patent in the second year of life, or if the patient experiences systemic complications of portosystemic shunting regardless of age, the consensus is that shunt closure is important. For extrahepatic CPSS, pre-emptive closure even in asymptomatic patients is the consensus, as spontaneous closure is unlikely, extrahepatic shunts are associated with more severe complications, and the severest cases require liver transplantation [5,28,29,30]. In this study, 8 of the 13 cases that underwent surgical treatment were asymptomatic (Table 2). Early detection is clearly a key factor in the ability to provide strict follow-up and accurate treatment. That is, if a high probability of CPSS can be accurately recognized at NBS, the prognosis will be improved.

One report from Japan used screening items and confirmatory blood tests to detect CPSS, as in this study [8]. The small number of cases in that study made generalization of the results difficult. In this study, we were able to set the sensitivity and specificity to construct a prediction model by increasing the number of cases analyzed and extending the observation period. However, these data were only from Saitama Prefecture and represent approximately one-sixteenth of the Japanese population. Gal-1-P/Gal was collected on days 4 to 6 after birth, while TBA and NH3 were collected from day 14 to two months after birth. In other words, this study combined values from two different periods. This difference in the timing of blood collection may have led to some cases of physiological PDV being classified as transient. Murayama et al. [31] reported that functional closure of the ductus venosus occurs at 10.2 days old in babies with 29–32 weeks of gestation, 7.1 days old in babies with 33–36 weeks of gestation, and 4.6 days old in babies with 37–41 weeks of gestation. Considering that most of our cases were full-term, although about half of the cases showed a patent ductus venosus at the time of DBS collection, we believe that functional closure had been achieved by the time of the confirmation test. In the future, the effects of physiological PDV could be resolved by performing DBS collection and confirmatory testing at the same time.

By combining the three items (Gal-1-P/Gal, TBA, and NH3), we were able to establish a cut-off value that can be used to predict CPSS from an early stage. Further, the ROC was ≥0.8 (Figure 2), which can be considered to reflect excellent discrimination performance [32]. CPSS is reportedly generally suspected if TBA and NH3 levels are elevated [33,34,35]. In this study, we were able to create a useful model for predicting CPSS through combining these factors. In fact, Case 12 (Table 2) was considered for shunt closure while asymptomatic based on our prediction model (Figure 2 and Figure 3). Sensitivity and specificity could be easily adjusted to desired values that would increase the positive predictive value (Table S1).

Despite setting the cut-off value to give the best sensitivity and specificity, six CPSS cases showed false-negative results (Figure 3). Five of these six cases had PDV and high initial Gal-1-P levels. In three of the four PDV cases, the ductus venosus closed within five months. The last false-negative case (Case 6 in Table 2) had an extrahepatic shunt and required surgical treatment. We consider that Case 6 (Table 2) was due to high Gal-1-P and low TBA levels, but the reason for this remains a cause for concern.

Unfortunately, no significant differences in P were identified between the surgical and spontaneous closure cases (*p*-value = 0.35). This means more reliable results from a large-scale study are needed.

This study only included data from two facilities in Saitama Prefecture. In the future, we hope that a large-scale prospective cohort study will be conducted that also includes the timing of sample collection, leading to the construction of a more reliable predictive model. The present study has shown that this goal is feasible.

## 5. Conclusions

When the cut-off for the predicted probability of disease was P > 0.357 using Gal-1-P/Gal, TBA, and NH3, CPSS was able to be identified with 86.4% sensitivity (95%CI 72.6–94.8%) and 81.9% specificity (95%CI 72.0–89.5%). Using this predictive model, we might be able to detect CPSS early, leading to early intervention.

## Figures and Tables

**Table 1:** Final diagnosis of analyzed patients.

Analyzed Patients (*n* = 153)	
**Final Diagnosis**	***n* (%)**
Enzyme deficiency	18 * (11.8%)
Portosystemic shunt	44 (28.8%)
Intrahepatic	32
Extrahepatic	12
Citrin deficiency	7 (4.6%)
Transient galactosemia	83 (54.2%)
Other	1 (0.6%)

* *GALT* heterozygous, 2 cases; *GALK*, 2 cases; *GALE*, 11 cases; *GALM*, 3 cases.

**Table 2:** Characteristics of patients with surgical interventions.

Case	Type of Shunt	Anatomy	Clinical Features	Treatment	Gal-1-P/Gal	TBA (μmol/L)	NH3 (μg/dL)	P
1	extrahepatic	IMV-LFV	no symptoms	endovascular	2.73	56.7	90.00	0.51
2	extrahepatic	PV-LRV	no symptoms	endovascular	1.25	47.2	132.00	0.67
3	extrahepatic	absence of PV	no symptoms	LT	0.62	20.0	90.26	0.43
4	extrahepatic	hypoplastic PV, SMV-LHV	MR	endovascular	1.69	26.6	95.37	0.45
5	extrahepatic	absence of PV, SMV-azygos vein	unclear	unclear	0.80	80.0	115.80	0.74
6	extrahepatic	SV-LRV	no symptoms	endovascular	3.97	28.3	68.12	0.30
7	extrahepatic	SV-LRV	MR	endovascular	1.98	72.6	76.64	0.54
8	extrahepatic	hypoplastic PV, SV-LRV	Hypermanganesemia, hepatic atrophy	surgical	0.70	77.4	109.00	0.71
9	extrahepatic	PV-RA	no symptoms	surgical	2.88	114.8	137.94	0.84
10	extrahepatic	SV-LRV	no symptoms	surgical	1.67	68.0	91.96	0.59
11	intrahepatic	PDV	heart failure with COA	operation for COA	0.80	137.2	68.00	0.76
12	intrahepatic	PV-LHV	no symptoms	endovascular	1.69	150.6	102.00	0.86
13	intrahepatic	PDV	no symptoms	endovascular	0.69	12.6	84.00	0.38

Gal-1-P, galactose-1 phosphate; Gal, galactose; TBA, blood total bile acid; NH3, ammonia; IMV, inferior mesenteric vein; FV, femoral vein; PV, portal vein; RV, renal vein; SMV, superior mesenteric vein; RA, right atrium; PDV, patent ductus venosus; SV, splenic vein; HV, hepatic vein; COA, coarctation of the aorta; MR, mental retardation; LT, liver transplantation.

**Table 3:** Clinical characteristics of patients with portosystemic shunt and temporary galactosemia.

	CPSS (*n* = 44)	Transient Galactosemia (*n* = 83)	*p*-Value
Birth weight, g, mean (SD)	2887.4 (422.0)	2988.7 (409.1)	0.19
Sex ratio, male/female	24:20	52:31	0.45
Galactose, mg/dL, median (IQR)	5.65 (4.00–9.58)	2.80 (1.20–4.60)	<0.001
Total galactose, mg/dL, mean (SD)	12.95 (5.48)	17.70 (6.75)	<0.001
Gal-1-P, mg/dL, median (IQR)	7.20 (3.67–11.40)	21.30 (8.64–27.80)	<0.001
Gal-1-P/Gal, median (IQR)	1.05 (0.61–1.95)	7.69 (1.34–20.90)	<0.001
ALT, U/L, median (IQR)	18.00 (14.00–26.75)	18.00 (14.00–22.00)	0.37
Albumin, g/dL, mean (SD)	3.63 (0.35)	3.80 (0.34)	0.009
Ammonia, μg/dL, median (IQR)	90.13 (68.03–118.36)	68.12 (54.50–86.00)	0.002
Prothrombin time, % median (IQR)	86.00 (73.55–94.90)	89.00 (83.00–96.30)	0.07
Total bile acid, μmol/L, median (IQR)	53.50 (28.75–79.90)	17.90 (11.00–41.20)	<0.001
Direct bilirubin, mg/dL, median (IQR)	0.45 (0.20–0.80)	0.40 (0.20–0.60)	0.46

Reference ranges derived from the institutional laboratory standards: ALT (M: 10–42 U/L, F: 7–23 U/L); albumin: 4.1–5.1 g/dL; ammonia: 12–66 μg/dL; prothrombin time: 74–120%; total bile acid: 0–10 μmol/L; direct bilirubin: 0–0.4 mg/dL.

**Table 4:** Crude and adjusted odds ratios according to logistic regression analysis.

	Crude OR	95%CI	Adjusted OR	95%CI	Adjusted OR	95%CI	*p*-Value
			**(Enter)**		**(Selected Variables)**		
Birth weight, g	1.00	0.99–1.00	1.00	0.99–1.01			
Sex, male	0.72	0.34–1.51	0.53	0.18–1.50			
Gal-1-P/Gal	0.87	0.80–0.94	0.90	0.83–0.97	0.90	0.83–0.97	<0.001
ALT, IU/L	1.03	0.99–1.07	1.03	0.97–1.10			
Albumin, g/dL	0.23	0.07–0.71	0.37	0.08–1.72			
Ammonia, μg/dL	1.02	1.00–1.04	1.02	1.00–1.04	1.02	1.00–1.04	0.03
Prothrombin time, %	0.98	0.95–1.01	0.96	0.93–1.00			
Total bile acid, mg/dL	1.02	1.01–1.04	1.02	1.00–1.04	1.02	1.00–1.03	0.01
Direct bilirubin, mg/dL	1.84	0.73–4.62	0.48	0.13–1.67			

## Data Availability

The original contributions presented in this study are included in the article/Supplementary Material. Further inquiries can be directed to the corresponding author.

## Supplementary Materials

The following supporting information can be downloaded at https://www.mdpi.com/article/10.3390/ijns11030061/s1. Table S1: Date of biomarkers before and after shunt closure. Table S2: Sensitivity and specificity of predicted disease probability (P). Figure S1: Dot plot showing three parameters (Gal-1-P/Gal ratio, total bile acid, ammonia), separately. Figure S2: Predicted probability (P) for cases diagnosed with ultrasound alone (a), and with both contrast-enhanced CT and ultrasound (b).
