# Supplementary material for: Prediction of Congenital Portosystemic Shunt in Neonatal Hypergalactosemia Using Gal-1-P/Gal Ratio, Bile Acid, and Ammonia
Source: Int J Neonatal Screen. 2025 Aug 7;11(3):61. doi: 10.3390/ijns11030061 (PMC12372138; doi:10.3390/ijns11030061)
Supplement: Supplementary file 1 [file IJNS-11-00061-s001.zip › IJNS-3749829 Figure S1.pptx]

## Slide 1
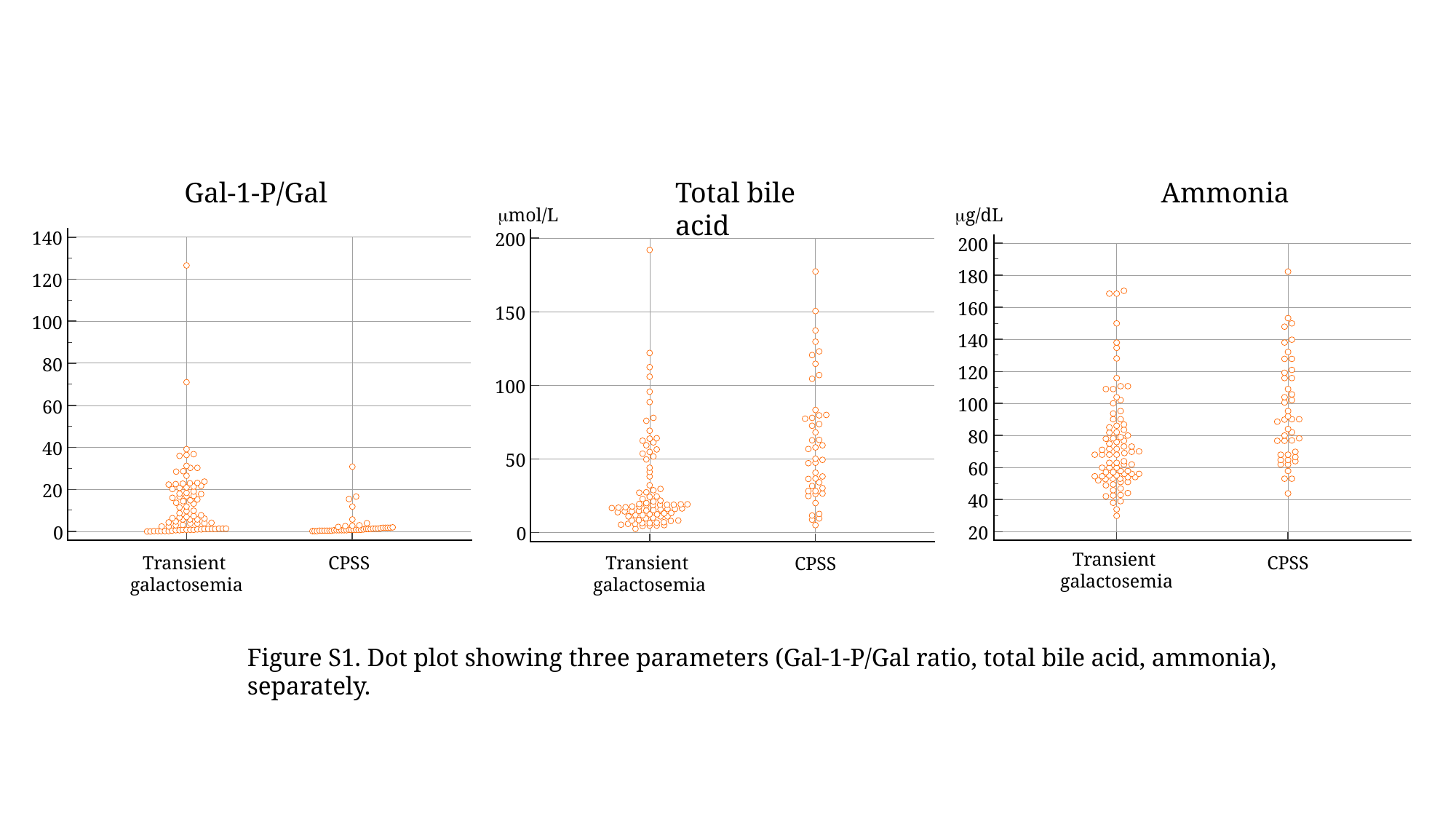

Ammonia
Gal-1-P/Gal
Total bile acid
μmol/L
μg/dL
140
120
100
80
60
40
20
0
CPSS
Transient
galactosemia
200
150
100
50
0
200
180
160
140
120
100
80
60
40
20
Transient
galactosemia
CPSS
Transient
galactosemia
CPSS
Figure S1. Dot plot showing three parameters (Gal-1-P/Gal ratio, total bile acid, ammonia), separately.
