# Supplementary material for: Prediction of Congenital Portosystemic Shunt in Neonatal Hypergalactosemia Using Gal-1-P/Gal Ratio, Bile Acid, and Ammonia
Source: Int J Neonatal Screen. 2025 Aug 7;11(3):61. doi: 10.3390/ijns11030061 (PMC12372138; doi:10.3390/ijns11030061)
Supplement: Supplementary file 1 [file IJNS-11-00061-s001.zip › IJNS-3749829 Figure S2.pptx]

## Slide 1
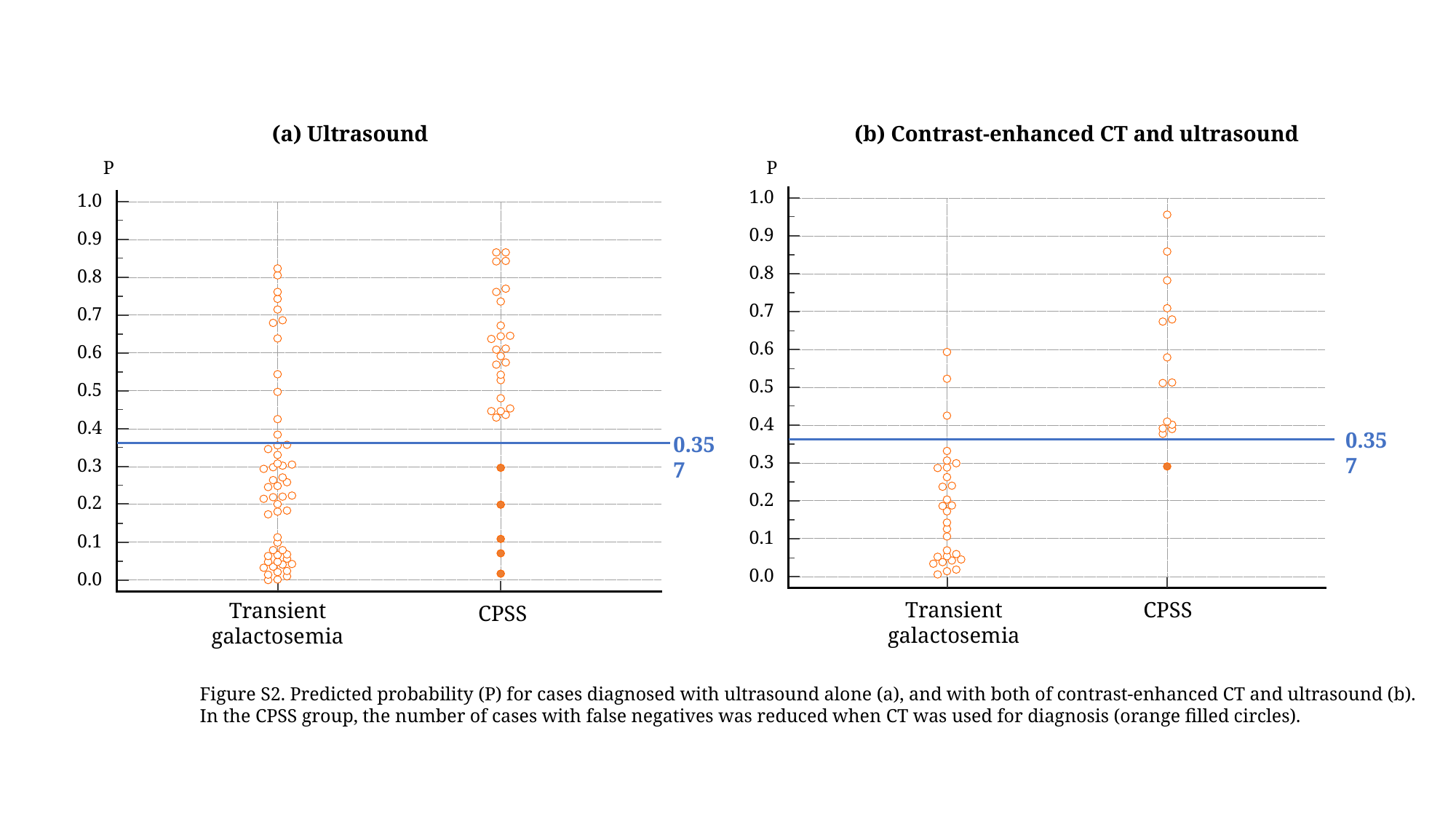

(a) Ultrasound
(b) Contrast-enhanced CT and ultrasound
P
P
1.0
0.9
0.8
0.7
0.6
0.5
0.4
0.3
0.2
0.1
0.0
1.0
0.9
0.8
0.7
0.6
0.5
0.4
0.3
0.2
0.1
0.0
0.357
0.357
CPSS
Transient
galactosemia
Transient
galactosemia
CPSS
Figure S2. Predicted probability (P) for cases diagnosed with ultrasound alone (a), and with both of contrast-enhanced CT and ultrasound (b).
In the CPSS group, the number of cases with false negatives was reduced when CT was used for diagnosis (orange filled circles).
