# Supplementary material for: Prediction of Congenital Portosystemic Shunt in Neonatal Hypergalactosemia Using Gal-1-P/Gal Ratio, Bile Acid, and Ammonia
Source: Int J Neonatal Screen. 2025 Aug 7;11(3):61. doi: 10.3390/ijns11030061 (PMC12372138; doi:10.3390/ijns11030061)
Supplement: Supplementary file 1 [file IJNS-11-00061-s001.zip › IJNS-3749829 Table S1.pdf]

Table S1. Data of biomarkers before and after shunt closure.

	**Before shunt closure**				**After shunt closure**	
**Case**	Gal-1-P /Gal	TBA (µmol/L)	NH3 (µg/dL)	P	TBA (µmol/L)	NH3 (µg/dL)
**1**	2.73	56.7	90	0.65	unclear	unclear
**2**	1.25	47.2	132	0.83	6.6	44.0
**3**	0.62	20.0	53	0.38	19.0	44.3
**4**	1.69	26.6	56	0.39	3.0	35.8
**5**	0.80	80.0	68	0.66	9.0	64.7
**6**	3.97	28.3	40	0.25	3.0	29.0
**7**	1.98	72.6	45	0.47	8.0	23.8
**8**	0.70	77.4	109	0.83	6.4	36.0
**9**	2.88	114.8	81	0.77	4.1	35.8
**10**	1.67	68.0	54	0.52	6.0	32.4
**11**	0.80	137.2	68	0.81	unclear	unclear
**12**	1.69	150.6	102	0.91	5.8	39.0
**13**	0.69	12.6	84	0.52	8.4	28.0
